# Anthropomorphic motion planning for multi-degree-of-freedom arms

**DOI:** 10.3389/fbioe.2024.1388609

**Published:** 2024-05-28

**Authors:** Xiongfei Zheng, Yunyun Han, Jiejunyi Liang

**Affiliations:** ^1^ State Key Laboratory of Intelligent Manufacturing Equipment and Technology, Huazhong University of Science and Technology, Wuhan, China; ^2^ Department of Neurobiology, School of Basic Medicine, Tongji Medical College, Huazhong University of Science and Technology, Wuhan, China

**Keywords:** anthropomorphic, motion planning, arms, motion redundancy, motion variation, motion coordination

## Abstract

With the development of technology, the humanoid robot is no longer a concept, but a practical partner with the potential to assist people in industry, healthcare and other daily scenarios. The basis for the success of humanoid robots is not only their appearance, but more importantly their anthropomorphic behaviors, which is crucial for the human-robot interaction. Conventionally, robots are designed to follow meticulously calculated and planned trajectories, which typically rely on predefined algorithms and models, resulting in the inadaptability to unknown environments. Especially when faced with the increasing demand for personalized and customized services, predefined motion planning cannot be adapted in time to adapt to personal behavior. To solve this problem, anthropomorphic motion planning has become the focus of recent research with advances in biomechanics, neurophysiology, and exercise physiology which deepened the understanding of the body for generating and controlling movement. However, there is still no consensus on the criteria by which anthropomorphic motion is accurately generated and how to generate anthropomorphic motion. Although there are articles that provide an overview of anthropomorphic motion planning such as sampling-based, optimization-based, mimicry-based, and other methods, these methods differ only in the nature of the planning algorithms and have not yet been systematically discussed in terms of the basis for extracting upper limb motion characteristics. To better address the problem of anthropomorphic motion planning, the key milestones and most recent literature have been collated and summarized, and three crucial topics are proposed to achieve anthropomorphic motion, which are motion redundancy, motion variation, and motion coordination. The three characteristics are interrelated and interdependent, posing the challenge for anthropomorphic motion planning system. To provide some insights for the research on anthropomorphic motion planning, and improve the anthropomorphic motion ability, this article proposes a new taxonomy based on physiology, and a more complete system of anthropomorphic motion planning by providing a detailed overview of the existing methods and their contributions.

## 1 Introduction

Robots, following meticulously calculated and planned trajectories, have been providing safer and more efficient working environments for humans with superior quality in many scenarios. Especially in industrial manufacturing, robotic arms can even independently perform various tasks such as handling, machining, and assembling in specified conditions, which dramatically improves the productivity.

However, conventional motion planning techniques typically rely on predefined algorithms and models that may not be adaptable to new environments. Especially when faced with the increasing demand for personalized and customized services, predefined motion planning cannot be adjusted in time to adapt to personal behavior, which will seriously affect the efficiency of task completion. In this case, robots need to establish a stronger connection with humans by increasing interaction and expanding the human-robot sharing space in order to develop algorithms and models that could adapt to the individual preferences, habits, and needs.

Recent researches demonstrated that humans are more inclined to accept actions similar to themselves during human-robot interactions (Arkin and Moshkina, 2014; Dragan and Srinivasa, 2014). To meet this requirement, researches across the world have been initiated to improve human-robot interaction by enhancing the anthropomorphism of robot motion (Kiesler et al., 2008; Kühnlenz et al., 2013). There are three main scenarios, service robots, new industrial robots, and wearable robots (exoskeletons), where anthropomorphism of robot arm motion is highly demanding and the robots need to interact and collaborate with humans in a shared human-robot interaction space. For anthropomorphic service robots, adopting anthropomorphic motion can significantly enhance the robot’s similarity to humans, foster a greater sense of familiarity, and thus increase the robot’s acceptance among users. For new industrial robots, anthropomorphic motion can enhance not only the synchronicity between the workers and the robots during collaborative process, but also the efficiency and overall safety of the human-robot interaction. During the interaction, the workers can accurately and promptly comprehend the robots’ behavior, which enables them to make rational assumptions about the robots’ motion patterns. When there is a risk of collision between a robot and a worker or the environment, the worker can take prompt action to avoid collisions and increase the safety. In addition, anthropomorphic motion provides a better way to interact, which greatly reduces the training time of the worker. For wearable robots, anthropomorphic motion has a more direct impact on the therapeutic performance of rehabilitation training. For exoskeletons used to enhance human function, if the motion does not match the way that the patients move, the rehabilitation training will not only fail to enable the patient to regain movement ability, but may cause secondary damage to the patient.

How to achieve anthropomorphic motion in robots? An analysis of human movement shows that the process of human movement at the physiological level can be represented by the process chain: neural commands-muscle activation-joint motion-hand movement-task goal (Flash et al., 2013). Inspired by this chain, current researches on anthropomorphic motion mainly focus on three directions: anthropomorphic structural design, anthropomorphic trajectory generation, and anthropomorphic motion control (Kulic et al., 2016).

In anthropomorphic structural design, researchers have developed humanoid robots that closely resemble humans in appearance, joint structure, and motion by modeling the human musculoskeletal system. This kind of design is inspired by biology and based on research in human anatomy, kinesiology, and biomechanics (Ogawa et al., 2011; Paik et al., 2012; Lenzi et al., 2016), which has contributed to an increased acceptance and trust among users. However, these robots are still challenged in mimicking the flexibility, elasticity and stability of the human limbs.

In anthropomorphic trajectory generation, researchers tried to explore the human upper limb movement laws from the motion posture and trajectory, combine it with human kinematics and physiology, and determine the optimal motion trajectories and movement sequences through simulation and experimental validation, so as to make the robot’s motion more natural, smooth, and match the physiological characteristics of the human. Specifically, by studying the correlations and variations between the rotation angles of certain joints (e.g., elbow elevation angle (Kim et al., 2006)) and hand postures, researchers have generated anthropomorphic motion for robotic arms (Zanchettin et al., 2013; Su et al., 2018). In addition, the researchers found some motion characteristics, such as bell-shaped velocity curve (Ferrer et al., 2023), sinusoidal acceleration curve (Morasso, 1981), bell-shaped positional variance (Taniai et al., 2022), Fitts’s Law (Fitts, 1954), and temporal distribution (Young et al., 2009), for analyzing physical quantities, such as joint velocities, accelerations, and trajectories during the natural movement of the upper limbs, and used them as criteria for generating anthropomorphic trajectories for robotic arms. However, these studies only focus on the kinematic nature of upper limb movement, and have not tapped into the cornerstones of upper limb movement laws that underlie anthropomorphic motion generation in robotic arms. Guigon et al. (2007) assumed that the motor control is governed by four principles (separation principle, optimal feedback control principle, maximum efficiency principle, constant effort principle) by building a computational model, and attempted to provide a unified explanation of biological motor behavior. von Zitzewitz et al. (2013) argued that robot perception plays a crucial role in human-robot interaction, and anthropomorphism as a factor of interaction efficiency should not be considered as a single parameter, but as a variable influenced by other parameters. They proposed to divide the network of parameter fields describing anthropomorphism into two categories: appearance and behavior (Minato et al., 2012) to describe the static and dynamic states of the robot, respectively. However, these motion parameters only describe possible similar aspects of robots and humans from multiple perspectives, but do not provide quantitative anthropomorphic metrics that can be directly used as criteria for generating anthropomorphic motion.

In anthropomorphic motion control, researchers are trying to explore how to achieve precise control and adaptive regulation of robot motion by mimicking human movement styles and behavioral characteristics, so that robot motion will have similar motor capabilities to those of humans, which includes accurate collection and processing of sensor data, as well as real-time adjustment and optimization of control algorithms. Most approaches rely on high-gain control and fast control loops that enable robots to perform specific tasks in structured environments, but are unable to deal with unexpected disturbances or system variations, and do not simulate the flexibility, versatility, and robustness of human movement control.

Among the three research directions, anthropomorphic trajectory generation can provide input for anthropomorphic motion planning based on human motion characteristics, which is crucial for robots to realize anthropomorphic motion. It enables robots with natural and smooth motion, enhances their adaptability and safety, and improves the performance of human-robot interaction so that robots have more anthropomorphic motion and behavior characteristics. Overall, current studies have made some progress in improving the anthropomorphism of robot motion, but there is still no consensus on the criteria by which anthropomorphic motion is accurately generated. The main reason is the criteria derived from existing research may not be able to fully cover the most important aspects concerning the similarity between robots and humans.

In recent years, researchers have gradually deepened the study of anthropomorphic motion planning and applied it to humanoid robots, which has made considerable progress. Service robots have gradually been a part of people’s daily lives, cooperating with them in a friendly way (Potkonjak et al., 2001). New industrial robots can not only work closely with human workers to perform complex manufacturing and assembly tasks, but can also operate independently in harsh environments such as high temperatures and pressures, increasing the efficiency of industrial production and ensuring worker safety (Zacharias et al., 2011). Wearable robots enhance or reconstruct the natural movement of disabled limbs (Soltani Zarrin et al., 2021). These products dramatically improve efficiency and deliver better care and services that not only improve quality of life, but also drive technological advancement and innovation. At the same time, biomechanics, neurophysiology, and exercise physiology have advanced our understanding of the body’s mechanisms for generating and controlling movement, and upper limb motion patterns were progressively resolved, which provides a physiological basis for motion characteristic extraction. With the help of tools in statistics and computer graphics, researchers can discover the laws embedded in the upper limb movement data (or movement sequences), extract the motion characteristics, describe them intuitively and quantitatively, realistically show the upper limb movement status and motion characteristics, build models to describe human movement behaviors, so as to replicate human movement on a humanoid robot as closely as possible through motion planning. In addition, the increasing computational capability of motion models has facilitated the continuous improvement of motion control schemes, which in turn has promoted in-depth exploration of the nature of motion. At the same time, concepts such as the “spatiotemporal characteristics” inherent in the movement process have been proposed as new anthropomorphic evaluation criteria. With the development of virtual reality, machine learning, intent recognition, semantic grasping, and other related technologies, motion accuracy has been significantly improved, which has driven the emerge of new anthropomorphic motion planning methods to some extent.

However, the current anthropomorphic motion planning algorithms still have some problems in practical applications, that need to be further improved and solved. First, the modeling of the biomechanical characteristics of the human movement is not investigated enough. Anthropomorphic motion planning algorithms are often based on simplified models of human biomechanics, ignoring many details and complexities, which can result in the differences between the movements of robots and human, and the lack of biomechanical naturalness. As a result, biomechanical characteristics such as human bones, muscles, and joints must be more accurately and meticulously modeled to improve the realism and fidelity of robotic motion. Second, there is a lack of understanding of human movement variation. Human upper limb movement has some individual variation and can vary considerably from person to person. Moreover, unlike the lower limbs, the upper limbs do not have a single, periodic functional activity, which makes it difficult to establish a standardized experimental paradigm for the upper limbs. However, current anthropomorphic motion planning algorithms are typically modeled based on average motion data or data from a small number of subjects, ignoring the individual variation, which leads to a lack of personalization and diversity in robot motion. Third, there is a lack of in-depth research on neurophysiology and exercise physiology. Human upper limb movement involves the coordination of multiple neuromuscular systems and complex neural signaling control processes. However, current anthropomorphic motion planning algorithms have an insufficient understanding of these neurophysiological and exercise physiological mechanisms and lack detailed modeling and simulation of neuromuscular models and motor control signals. Therefore, further in-depth studies of neurophysiology and exercise physiology are needed to incorporate these physiological characteristics into anthropomorphic motion planning algorithms to improve the biomimicry and realism of motion. Fourth, the problem of motion planning and obstacle avoidance in complex environments has not been fully solved. In practical applications, robots often need to plan their motion and avoid obstacles in complex, dynamic environments. The potential failures coming from the unpredictability of robot-human interactions still troubles the users, which seriously hinders the large-scale application of humanoid robots. Further research is needed on how to generate adaptive and flexible anthropomorphic motion that take into account environmental constraints. This may involve the integration of perception, planning, and control, as well as accurate modeling and real-time updating of environmental information. Fifth, current humanoid robots still have limited autonomy and adaptability. Most anthropomorphic motion planning algorithms (including inverse kinematics methods (Li G. et al., 2019; Li et al., 2022), visual teaching (Kuniyoshi et al., 1994), reference path generation for upper limb rehabilitation exoskeletons (Soltani-Zarrin et al., 2017), optimal control methods (Taïx et al., 2013; Geoffroy et al., 2014), etc.) rely on offline planning and must be pre-programmed or rely on external commands to perform the task, and are simply not capable of continuously performing complex tasks outside of a specific work environment. Sixth, the complexity of the human body’s own movement laws leads to the fact that a single (or several) anthropomorphic criterion is still unable to describe most of the human movements, resulting in a lack of anthropomorphism in robot motion, which is far from natural human movement behavior. Therefore, the existing anthropomorphic motion planning methods are still not sufficient for practical applications.

Despite the existence of articles that provide a cursory overview of classification methods for anthropomorphic motion planning of robotic arms, including sampling-based (random search), optimization-based (constrained optimization), and imitation-based (demonstration learning) approaches, these overviews typically rely on simple distinctions based on the nature of the planning algorithms. However, they lack a systematic examination of the rationale behind the extraction of upper limb motion characteristics using these methods. Furthermore, these characteristics do not comprehensively capture the full range of upper limb motion patterns and fail to elaborate on their specific roles in the development of an anthropomorphic motion planning framework. Some research has revealed the existence of invariant motion characteristics in the natural movement of human upper limbs (Soechting and Lacquaniti, 1981; Atkeson and Hollerbach, 1985), which contribute to the uniqueness of human motion behavior and its difficulty to emulate or replicate. Despite the general similarity in morphological structure and motion patterns between current humanoid robotic arms and human upper limbs, the lack of comprehensive guidance from human upper limb movement laws prevents the achievement of highly anthropomorphic motion.

**FIGURE 1:**
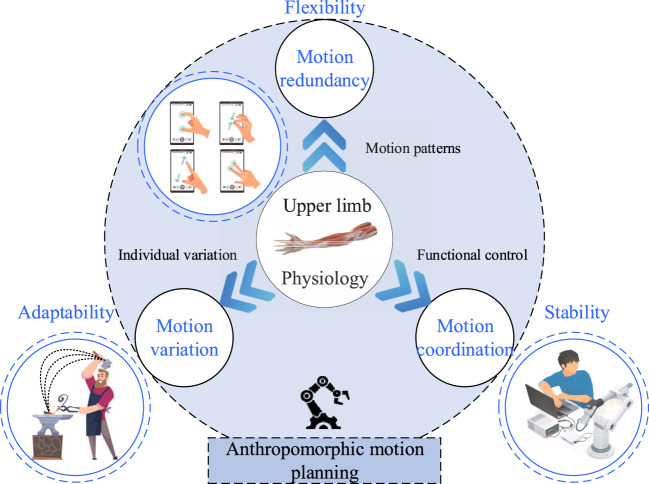
A frame of anthropomorphic motion planning system composed of three components.

To facilitate the realization of more natural anthropomorphic motion in humanoid robotic arms, the key milestones and most recent literature have been collated and summarized, and three essential conditions have been identified. These are: 1) Motion redundancy. It is crucial for achieving the flexibility and accuracy of human upper limb movement through different motion patterns, serving as the foundation for humans’ robust motion capabilities and interactive abilities. 2) Motion variation. It accounts for the diversity and individual variation in human upper limb movement, representing a unique capacity for adaptation, self-learning, and continuous evolution. 3) Motion coordination. It ensures the efficiency and stability of human upper limb movement by functional control, providing a safeguard for generating and controlling motion while maintaining inertia. These three characteristics are interrelated and interdependent, as shown in Figure 1, posing a challenge for the anthropomorphic motion planning framework. Therefore, this article systematically analyzes the anthropomorphic motion planning methods in recent years, with a particular focus on the concepts of motion redundancy, motion variation and motion coordination, and discusses the limitations and challenges.

## 2 Motion redundancy

Humans can adjust the posture of the upper limbs according to the position of the target to perform the task with appropriate movements, such as surgeons operating on small wounds according to different circumstances, “cutting”, “suturing”, “knotting” and others to ensure the success of the operation, all of which rely on the flexibility provided by the redundancy of the upper limbs. The redundancy of the upper limbs provides humans with a wealth of motor skills, adaptations, and means of perceptual communication that enhance their ability to interact and adapt with others and the environment.

In practical scenarios, robotic arms not only have to interact with humans, but also have to take into account obstacle avoidance and joint limitations. A common method for generating anthropomorphic motion trajectories is to preplan the collision-free waypoints of the end-effector in Cartesian space using path planning algorithms such as PRM (Kavraki et al., 1996), RRT (Kuffner and LaValle, 2000), and CHOMP (Zucker et al., 2013), and then establish a mapping relationship between Cartesian space and joint space, and solve for the joint angles of the robotic arm at different moments by inverse kinematics. The trajectories of each joint are generated by interpolation. Commonly used interpolation methods are cubic polynomials, quintic polynomials, and spline curves (Xie et al., 2011; Hu et al., 2023; Li et al., 2019; Wang et al., 2020). The key to this approach is to develop an appropriate kinematic/dynamical model for the upper limbs to solve the inverse kinematics.

In studies, human upper limbs are often regarded as an articulated structure composed of connecting rods (bones) and joints with a high degree of redundancy, where different joints cooperate in a variety of combinations to perform complex tasks according to different needs (Kulic et al., 2016; Wei and Zhao, 2019). It is generally accepted that the human upper limbs have 10 DoFs, while the 10-DoFs robotic arm is too flexible to control (Liu and Xiong, 2013). For simplicity, a simplified 7-DoFs robotic arm with only three joints: shoulder, elbow, and wrist, was as a sphere-revolute-sphere structure (Xia et al., 2021), which was roughly the same as the human upper limb in shape and motion style, and has become the mainstream approach. This kind of anthropomorphic design is the basis for realizing human-like behaviors (Fang et al., 2019). Meanwhile, a study proposed the use of Rapid Upper Limb Assessment (RULA) to evaluate the naturalness of the humanoid robotic arm configuration (Zacharias et al., 2011). However, the human-like appearance and configuration of a robotic arm alone is not enough to generate anthropomorphic motion. The reason is the generation mechanism of natural human movement is still not fully revealed, that is, how humans deal with redundancy in the upper limbs during complex motion. From a physiological perspective, the redundancy of the human upper limbs is primarily attributable to the central nervous system’s ability to control the contraction and relaxation of muscle groups through intricate neural networks and signaling pathways. This control mechanism enables the precise control of multiple muscles corresponding to multiple joints, thereby facilitating the precise control of multiple joints in the upper limbs. The problem of redundancy in humanoid robotic arms exists at the level of kinematics and dynamics, which leads to an infinite number of inverse kinematics solutions. How to solve the inverse kinematics problem and find the best solution that fits the configuration of the robotic arm among countless solutions is one of the difficulties in anthropomorphic motion planning.

Overall, there are three typical redundancy problems in the human upper limbs: 1) Trajectory redundancy in Cartesian space. That is, for a given task, the hand has multiple realization paths with non-unique trajectories. 2) Trajectory redundancy in joint space. That is, given a time-varying motion trajectory of the hand, it is still not possible to uniquely determine the kinematic parameters such as direction, angle, and velocity of each joint over time. 3) Redundancy in joint muscle forces and moments. Even after the trajectories of the joints over time are determined, it is still not possible to uniquely determine the forces and moments exerted on the joints by each muscle. A plethora of studies have been conducted to investigate these issues. Based on the laws presented during the natural movement of the human upper limbs for classification, the prevailing methodologies can be broadly categorized into two main categories. The first category involves constraining the optimization of a cost function representing the natural motion characteristics. The second category leverages the unique relationship between the joints of the upper limbs presented in natural motion.

### 2.1 Constrained optimization

A robotic arm may face multiple constraints, such as environmental constraints, task constraints, and coordination constraints, as it performs various operational tasks in an unstructured scenario. These constraints increase the difficulty of solving inverse kinematics. There is an argument that the redundancy problem can be viewed as a constrained optimization problem (Tommasino and Campolo, 2017). Solving inverse kinematics is essentially solving a nonlinear optimization problem where the optimal solution can be obtained by minimizing the cost function as follows

$$\left\{\begin{array}{c} \min\;f\left(x\right)\text{ over }x\\ s.t.\;g_{i}\left(x\right)\leq 0\\ h_{j}\left(x\right)=0 \end{array}\right.$$ (1)

where f is a nonlinear function/cost function, x is the state vector of the robotic arm, that is, the values for all joint space. In motion planning, x is not a single state at a given moment, but all states along the entire planning path, that is, $x=\left[x_{0},\cdots ,x_{t},\cdots ,x_{T}\right]$ . g is an inequality constraint, including linear and nonlinear constraints, which aims to strictly control the feasibility of the trajectory of the robotic arm and ensure that the robotic arm does not collide with objects. h is the equation constraint, that is, the target assigned to the robotic arm.

The cost function is mainly derived from the laws of natural movement of upper limbs. The study of point-to-point reaching movement of the human upper limbs revealed two of the most important motion characteristics, straight paths and bell-shaped speed profiles (Flash and Hogan, 1985; Todorov, 2004). In (Arimoto and Sekimoto, 2006), these two characteristics were used as criteria to determine the degree of anthropomorphism of the robotic arm’s trajectories. However, the criteria may not be sufficient for complex upper limb movement such as manual dexterity tasks like writing with a pen, threading a needle, or carving with a knife (Shin and Kim, 2015). To solve this problem, by thoroughly studying the smoothness of the upper limb trajectories, Todorov and Jordan (1998) found that a significant acceleration can cause a shock or jolt in the movement process, and that the human body maintains a very small acceleration during movement to prevent self-injury of the musculoskeletal system, which led to the proposal of an optimization criterion that used the minimum acceleration as a cost function. Li et al. (2020) used the minimum potential energy as an optimization criterion to achieve precise control of the motion to reduce the magnitude of the potential energy change of the upper limb exoskeleton and ensure the smoothness of the motion trajectory. In addition, many studies also used other laws of motion as cost functions, such as minimum torque (Kang et al., 2003), minimum time (Tangpattanakul and Artrit, 2009), and minimum joint torque change (Wada et al., 2001). These cost functions were used to explain the principles of how humans generate natural movement, that is, the existence of invariant motion characteristics in the upper limbs that are independent of factors such as target, motion magnitude and direction, initial position, and external loads. These criteria chosen for the cost function were derived from the regular analysis of human upper limb trajectories, which represent the common characteristics of most upper limb movement, and were used in most studies to evaluate whether the trajectory is anthropomorphic or not.

However, the application scenarios of the above methods are limited to point-to-point movement (where the shoulder is assumed to be stationary during movement) and are not suitable for activities of daily living (scenarios where the center of the shoulder is moving in real time). Therefore, it has been suggested that a single cost function can only partially explain the anthropomorphism of the upper limb movement, which only works under special movement and cannot be applied to most scenarios. As a result, it does not provide enough flexibility to the robotic arm. The combination of several cost functions may be the solution to this problem (Berret et al., 2011).

By investigating the interaction between nonlinear muscle dynamics and control principles based on previous work, Wochner et al. (2020) argued that the human body follows a combination of independent and recognized criteria for optimality when controlling the upper limbs to generate optimal trajectories. They used the combined cost function of smoothness (to prevent damage to the musculoskeletal system itself), energy (to reduce energy consumption during movement), and internal force (necessary for human movement) as a new optimization criterion to reveal the contribution of human muscle dynamics in point-to-manifold motion, which in turn generates anthropomorphic trajectories. Based on this research, Albrecht et al. (2011) assigned weighting factors to different optimization criteria to combine them into a new cost function, and simplified the multi-objective optimization problem by adjusting the weight factors to balance the relationship between multiple objectives, thus finding the optimal anthropomorphic trajectory that matches the configuration of the robotic arm. For conciseness, a brief summary of constrained optimization methods is shown in Table 1.

**TABLE 1:** Approaches of constrained optimization.

Study	Task	Anthropomorphic criterion	Approach	Contribution
Todorov and Jordan (1998)	Complex arm movements	Maximum smoothness	Constrained minimum-jerk model	Stronger relationship between the path and the speed profile
Wada et al. (2001)	Point-to-point movements	Minimum commanded torque change	A prediction algorithm using the Euler-Poisson equation	Obtain the converged solution in a very short time
Kang et al. (2003)	Reaching movements	Minimum joint torque	Minimum-torque model	Determine arm configurations during normal and natural movements
Arimoto and Sekimoto (2006)	Reaching movements	Straight paths, bell-shaped speed profiles	Virtual spring-damper hypothesis	Resolve the ill-posedness of inverse kinematics
Tangpattanakul and Artrit (2009)	Simulation of consecutive via-points	Minimum time	Harmony search algorithm	Obtain the optimal interval time and reduce complication and time consuming
Albrecht et al. (2011)	Reaching-to-a-bar tasks	mechanical energy, joint smoothness	Inverse optimal control	Support the cost combination hypothesis
Shin and Kim (2015)	Reaching, grasping, moving an object	Compare the hand and elbow trajectories through the simulations and experiments	Lagrangian multiplier optimization method	Human-likeness depends on the purpose of given tasks
Li et al. (2020)	Path tracking	Minimal potential energy	Zeroing dynamics method	Track the desired motion path accurately
Wochner et al. (2020)	Point-to-manifold reaching movements	Smoothness, energy, internal force	Bayesian optimization	A mixed cost function replicates the behavior much better than single criterion

These approaches can accurately identify the optimal solution that satisfies the constraints through mathematical optimization techniques. However, they entail a significant computational burden, particularly when searching for the optimal solution in a high-dimensional space, which may result in high computational costs.

### 2.2 Special relationships between joints

In addition to optimization methods, dimensionality reduction is another idea for dealing with redundancy: explore the special relationships between the shoulder, elbow, and wrist in the natural motion of the upper limbs to reduce redundant DoFs so as to obtain optimal inverse kinematic solutions.

The complete movement process of the upper limbs can be regarded as a process quantity. Each moment in the process corresponds to the posture of the upper limb and can be considered as a state quantity. The solution of the inverse kinematics of redundant arms can be decomposed into a finite number of state quantities. In order to find a suitable state quantity to describe the upper limb posture, the concept of arm triangle was introduced in (Berman et al., 2008). In (Seraji, 1989), a plane consisting of the shoulder, elbow, and wrist joints is used to describe the posture of the upper limbs, and the upper limbs are free to rotate around the shoulder and elbow joints, respectively. Liu et al. (2016) proposed a wrist-elbow-in-line method based on the similarity of the kinematic structures of the human upper limb and the humanoid robotic arm. The method introduced the elbow and wrist joint positions as key positions and reduced redundancy by using them as end-effector orientation constraints of the robotic arm. The positions of elbow and wrist joints in Cartesian space were used as configuration parameters of the robotic arm, and the anthropomorphic configuration was obtained by inverse kinematic analysis. However, due to the different lengths and joint limitations of the human upper limb and the robotic arm, the robotic arm is unable to create an anthropomorphic configuration at all times, which makes it difficult to perform fully anthropomorphic motion throughout the workspace. Artemiadis et al. (2010) realized that the shoulder and elbow joints are more flexible than the wrist joint by observing human writing movements. They also found that the three joints of the shoulder, elbow, and wrist are highly interconnected to form a specific plane, and this plane is deflected during movement. The angle of rotation formed by the deflection is unique, and is defined as the elbow elevation angle (Kim et al., 2006). Then they used the constraint equation formed by the elbow elevation angle to reduce the redundancy to obtain the kinematic inverse solution, and then obtain the best anthropomorphic motion trajectory that meets the human posture. However, this method ignores the effect of wrist posture on upper limb movement. Zanchettin et al. (2011) improved this method by taking wrist posture into account and using least-squares cluster analysis to derive the relationship with elbow elevation. Kim et al. (2012) proposed an inverse kinematics-based rotation angle estimation algorithm by linearly combining two different rotation angles resulting from kinematic and dynamic constraints. The algorithm successfully reproduced the natural motion of the human upper limbs with an error of less than 5° compared to real human movement and can be applied to wearable exoskeleton robots. Su et al. (2019) used a new deep convolutional neural network to establish the mapping relationship between rotation angle and hand pose, which improves the accuracy and iteration speed of motion reconstruction with strong robustness. A brief summary of using special relationships between joints to solve motion redundancy listed in Table 2.

**TABLE 2:** Approaches of special relationships between joints.

Study	Task	Anthropomorphic criterion	Approach	Contribution
Kim et al. (2006)	Point-to-point hand motion	Elbow elevation angle	Response surface methodology	First propose a mathematical representation for characterizing human arm motion
Zanchettin et al. (2011)	Hand motion along a sphere	Swivel angle	Cluster and weighted least-square approach	Provide a repeatable and identifiable kinematic constraint
Kim et al. (2012)	Natural human arm movement	Swivel angle	Kinematic and dynamic constraint	Reproduces natural human arm movement with less than five degrees of estimation error
Liu et al. (2016)	Self-motion	Self-motion angle	Wrist-elbow-in-line method	Validated in practice and extended for obstacle avoidance
Su et al. (2019)	Swivel motion	Elbow angle	Deep convolutional neural network	Reduce online prediction time, noise robustness

In comparison to complex mathematical optimization methods, these methods may exhibit higher computational efficiency and be suitable for application scenarios with high real-time requirements. However, due to the complexity of human motion, a single natural motion relation may not be applicable to all types of redundancy problems, thus requiring customized designs for different problems.

The motion redundancy in the upper limbs is of great importance as a primary solution in anthropomorphic motion planning. A variety of inverse kinematics methods proposed by the researchers provide ideas for solving the redundancy problem, which greatly advance the development of anthropomorphic motion planning. However, there are still some problems with current methods: inverse kinematics solution methods in the joint space lack sufficient physiological basis and it is inadequate to ensure the variations in the motion process and vulnerable to interference.

## 3 Motion variation

Humans can accomplish the same task in different ways, for example, when a blacksmith repeatedly strikes an iron block, the trajectory of the strike is different each time, which suggests that there is no rigidly fixed pattern of repetitive movements. We call this phenomenon motion variation. A large number of studies have confirmed that kinematic variation is considered to be a control strategy for the human motor system and also an intrinsic characteristic of multi-degree-of-freedom limb motion (Latash et al., 2002).

Motion variation represents the diversity of human movement, which is the difference in control, motion patterns, and experience habits of different individuals. These differences include the pattern of muscle activity, the variation of joint angles, and the way of force application, which are indispensable for humanoid robots to realize anthropomorphic motion planning. How do humanoid robots exploit these differences in motion? Imitating and learning human movement may solve this problem.

It is a great idea to accurately reproduce human movement on a robot. A motion capture system can be utilized to gather data on natural human movement and construct a motion database. By comparing the end-effector trajectory with the database, the robot joint configuration and motion trajectories can be predicted (Yamane, 2020). The approach not only avoids the joint redundant, but also controls the grasping force and posture, and predicts the position and time of grasping. However, the large dataset may reduce the prediction efficiency. The human position is collected by the motion capture system in advance and then converted into joint angle information offline, then sent to the robot controller for execution, so that the robot’s movements are modeled after human movements, which is only applicable in weak interaction scenarios (Liu et al., 2012; Zuher and Romero, 2012). However, the limitation of these approaches is that they rely exclusively on pre-collected data that do not cover all possible human-robot interaction situations. Consequently, the robot’s responses may not be sufficiently flexible. Furthermore, since the robots’ movements are entirely derived from pre-existing human movements, they can only move in a repetitive manner, which greatly limits their use in humanoid robots that require frequent interaction with the outside world.

To address this issue, it is necessary to adopt a more flexible and adaptable approach to the movement of humanoid robots. In addition to replicating human movements, the robot must also learn the motion patterns of the human upper limbs, which enables robots to transfer human motor skills into their own systems in a straightforward manner and employ appropriate methods to replicate anthropomorphic motor trajectories. These trajectories exhibit similar or comparable motor characteristics to humans, making them suitable for practical applications.

The motion patterns of the human upper limbs are unique and rely on a strong learning capacity, which enables humans to adapt to complex and changing environments based on previous experience (Huang and Zhang, 2020). Even without prior knowledge, humans are still able to interact correctly with objects in the surrounding environment. (Nagahama et al., 2021). The variation and adaptability of humans are crucial in achieving effective motion in various situations. It has been a challenging problem to equip robots with learning and motor skills of humans. Researchers attempt to understand the laws governing upper limb movement at the physiological level and map human behavior patterns to robotic motion strategies.

Neurophysiological studies have shown that the natural movement of the human upper limbs can be decomposed into a large number of small movement units that can be combined in an orderly fashion to produce a variety of complex movement, which researchers call movement primitives (Giszter, 2015). In fact, research results from several fields has shown that human upper limb movement exhibits “primitive” properties at the level of brain motor cortex (Averbeck et al., 2002), kinematics (Rohrer et al., 2002), and dynamics (Mussa-Ivaldi and Bizzi, 2000). Therefore, the movement primitives can be regarded as the implicit embodiment of human motion characteristics, which cannot only explain the law governing upper limb movement and enhance the understanding of their own motion, but also serve as a carrier to transfer the movement law from the human upper limb to the humanoid robotic arm, so as to make its motion anthropomorphic.

Based on the human upper limb movement dataset, the researchers proposed a demonstration-learning-reconstruction method to extract the movement primitives. Firstly, they used human natural motion data as demonstration trajectories. Then, they constructed a learning model using statistical methods to encode them. Finally, they applied upper limb motion characteristics to the robotic arm motion to reconstruct similar behaviors. The most common learning models are Gaussian mixture model (GMM), Dynamic movement primitives (DMP), and hidden Markov model (HMM). Specifically, GMM has powerful coding and noise reduction capabilities and is often used to solve high-dimensional problems. Deng et al. (2020) proposed a strategy for learning human motor skills using GMM, which allowed robots to learn how to successfully perform fixed impedance-based tasks and achieve safe human-robot cooperation. DMP has strong adaptability and robustness. Lauretti et al. (2019) proposed a method to obtain joint space and Cartesian space anthropomorphic trajectories using DMP and extracted DMP parameters as motion characteristics to obtain obstacle avoidance trajectories via locally weighted regression, which was finally experimentally validated on a humanoid robotic arm LWR4+ (KUKA, Augsburg, Germany). HMM has strong predictive ability and can easily extract motion characteristics. Takano and Nakamura (2017) proposed a method for extracting motion data information using HMM, which could control the moments of all joints of a humanoid robot to achieve the desired contact force and overall motion. Furthermore, Zhang et al. (2020) proposed a new anthropomorphic motion control framework using GMM and DMP to learn the demonstration trajectories and generate the anthropomorphic motion trajectories, which was tested on a mobile service robot to prove its effectiveness.

The anthropomorphic motion generated by the above work are all simple reaching movement. There are also many studies that have reproduced complex movement. Pignat and Calinon (2017) used a hidden semi-Markov model (HSMM) to enable the robot to successfully assist humans in dressing. Koenig and Matarić (2016) used Bayesian networks to enable the robot to perform basic movement such as grasping and releasing. Mülling et al. (2013) and Calinon et al. (2010) developed table tennis robotic systems for anthropomorphic motion using mixture of motor primitives (MoMP), HMM and Gaussian mixture regression (GMR), respectively. Yi et al. (2022) developed an autonomous robotic grasping system using an imitation learning algorithm consisting of K-means clustering and DMP, which could be finely manipulated using a variety of machine learning methods, and proved its reliability through evaluation. There are also studies on improving individual algorithms or combining multiple algorithms to improve iterative efficiency and reproduction accuracy, such as task-parameterized GMM is used to learn the demonstration trajectory to obtain motion characteristics, which enables the robot to perform the dual-arm sweeping task smoothly (Silvério et al., 2015). However, the reference movement for demonstration learning relies on the richness of experimental data. When adding new sample data to the training model for training, it is common practice to retrain the original model after increasing the number of network layers or changing the structure, which consumes a lot of time. This problem is simplified by the broad learning system based on incremental learning principle (Huang and Zhang, 2020). In the broad learning system, even if new sample data is added, there is no need to retrain the existing structure and parameters even if new sample data is added. We only need to compute the added parameters and assign new computational weights to easily achieve incremental learning of input samples, characteristic nodes, and enhancement nodes.

Even as the dataset continues to grow, new questions also arise. Researchers expect that robots with human motor skills will also have the ability to understand and predict in the same way that humans do. Humans participate in interactions by predicting the behavior of others (Wenderoth et al., 2012), while the robot’s motion commands are issued by a controller, whose output commands are preset by a human input program, and the accuracy of the preset program commands affects the anthropomorphism of the movement trajectories to some extent. The anthropomorphic motion trajectories generated by demonstration learning are too dependent on the reference trajectory, which means that changes in the content of the demonstration may lead to different extracted movement primitives. Therefore, each trajectory iteration accumulates small prediction errors, which leads to the deformation of the robotic arm motion (Sasagawa et al., 2021). To overcome this problem and avoid the chance of the parameters of the upper limb model, Yang et al. (2021) combined the multiple characteristics of the human upper limb movement process, adopted the reward function, and used reinforcement learning to plan the anthropomorphic motion of the humanoid robotic arm, and verified the feasibility and validity of the robotic arm in anthropomorphic motion through experiments.

Furthermore, imitation-based motion planning algorithms commonly utilize motion datasets derived from demonstrative samples to create motion models. However, these models display limited generalization, thereby limiting their usability in unstructured scenarios. As a result, the motion variation of the upper limbs is compromised. Therefore, to enhance robots’ capacity to mimic human-environment interaction, it is crucial to enhance the generalization of these models. The main factors affecting the generalization ability are the unknown environment and the targets. Learning reference inputs through DMP algorithm and adaptive optimal admittance control method can effectively improve the robot’s ability to interact with unknown environment (Xue et al., 2022). Compared to other algorithms, the traditional DMP algorithm has excellent generalization and anti-interference capabilities (Gong et al., 2020). However, the limitation of this algorithm is that when the demonstration trajectory is learned, the trajectory characteristics represented by the basis functions are fixed. Even if the starting point and scaling factor are changed, the result is only a change in speed and the scaling of the trajectory, which cannot be applied to different complex tasks and environments. Although some studies have improved the DMP by adding constraints, the results are still unsatisfactory (Gams et al., 2014; Huang et al., 2019). Qian et al. (2020) proposed a hierarchical demonstration learning framework that combined symbolic and trajectory learning to improve a robot’s ability to adapt to new tasks and environmental changes. Lu et al. (2023) combined DMP with neural networks and admittance control to incrementally update the nonlinear function by adding new basis functions and weights to mimic the new trajectory, and finally experimentally demonstrated that the generalization of the trajectory was improved. Averta et al. (2020) used functional principal component analysis (fPCA) to extract functional principal components/basis functions (describing the motion variance of each joint trajectory at the time level) from human upper limb movement data, and argued that a general upper limb motion trajectory can be described as an ordered combination of a set of functional principal components, and that an anthropomorphic motion trajectory could be generated by optimizing the weights of these functional principal components.

In order to quantify the variation of human movement, Gielniak et al. (2013) adopted variance as a variable in the algorithm when studying anthropomorphic motion planning, and used variance as a measure of the motion variation, and concluded through experiments that highly constrained movement or body parts have less variance (or motion variability), which is basically the same as the intuitive feeling of human movement. Table 3 gives an overview of 12 approaches to solve motion variation.

**TABLE 3:** Approaches to solve motion variation.

Study	Task	Anthropomorphic criterion	Approach	Contribution
Calinon et al. (2010)	Hitting a ball with a table tennis racket	Movement primitives	HMM and GMR	Present and evaluate an approach to allow robots to acquire new skills
Mülling et al. (2013)	Striking movements in table tennis	Movement primitives	MoMP	Presented a framework that allows a robot to play table tennis with a human
Gielniak et al. (2013)	Mimicking performance	Spatiotemporal correspondence	Human-like and variance optimization	Present a quantitative metric for human-like motion
Koenig and Matarić (2016)	A set of basic actions	A series of actions with features	Bayesian networks	Presents a framework for lifelong robot task learning from demonstrations
Pignat and Calinon (2017)	Dressing task	Movement primitives	HSMM	Propose a method for efficient skill acquisition
Takano and Nakamura (2017)	Touching an object with the right hand	Synthesis of joint angle sequences	HMM	Propose a method for motion synthesis and force control
Lauretti et al. (2019)	Reaching and pouring task	Four performance indices	Hybrid Joint/Cartesian DMPs	100% in avoiding obstacles and high Cartesian accuracy
Deng et al. (2020)	Drawing specific lines	Movement primitives	GMM	Present a hierarchical control scheme for human-robot co-manipulation
Zhang et al. (2020)	Feeding meals to patients	Human activity recognition	Combine GMM with DMP	Propose a novel human-like control framework for the mobile medical service robot
Averta et al. (2020)	30 activities of daily living	A variety of movement parameters	fPCA	Embed synergies of human movements for robot motion generation
Yang et al. (2021)	Reaching motion	Feature variables of human arm	Reinforcement learning	Present a humanoid method, and verify humanization, feasibility, and effectiveness
Yi et al. (2022)	Grasping complex-shaped objects	Movement primitives	K-means clustering and DMP	Presents an autonomous grasping approach for complex-shaped objects

Most of the current methods to solve the problem of robot motion variation are demonstration learning. Although they can effectively reproduce human upper limb movement under specific environments and tasks, the generalization ability is weak and difficult to apply to complex scenarios. In addition, the data samples must be expanded to improve the accuracy of the motion, but the large number of operations severely affects the efficiency of the robot motion. Therefore, the study of motion variation from a statistical point of view alone remains deficient and needs to be synthesized in terms of the hypostasis of human movement.

## 4 Motion coordination

The human body is a complex bio-motor system, and each of its movement behaviors requires coordination inside and outside the body, that is, the ability of a system’s various joints, components, or systems to work together and cooperate with each other in the execution of complex actions or task, which enables the system to achieve the efficient and precise completion of the task. In particular, the upper limbs play a very important role in human life (the function of the upper limbs accounts for about 60% of the whole body), and almost all daily activities require some coordination between the upper limbs (Guiard, 1987), and the level of coordination directly affects human movement ability (Freitas et al., 2016).

To successfully perform a daily activity, the human body requires a number of sensory organs to process information and control upper limb movement, a process whose mechanism is not fully understood. In neurophysiology, there is evidence that the central nervous system is responsible for the vast majority of human movement. When confronted with different external stimuli, humans are always able to respond appropriately, which relies heavily on human sensorimotor modeling (Wolpert and Ghahramani, 2000). According to the model, there is a relationship between sensory inputs and motor outputs in the human body, in which the particular patterns present are likely to be the criteria for the generation and control of movement by the central nervous system, which provides a physiological basis for anthropomorphic motion planning for robotic arms.

Some studies have designed mechanical musculoskeletal structures that mimic the human upper limbs based on the musculoskeletal kinesiology, and have used control strategies involving internal force kinematics (Tahara et al., 2006) to reproduce muscle activities as closely as possible in a biological motion pattern (Northrup et al., 2001), which provides an achievable platform for anthropomorphic motion planning. On this basis, how to provide the humanoid robotic arm with highly anthropomorphic motion ability becomes a challenge.

When people interact with the outside world, the whole process from contacting information to making a response is about 0.2–0.4 s (Otaki and Shibata, 2019). It remains an unsolved question how humans can easily coordinate multiple redundant DoFs of the body in a short period of time during movement. The causes of motion coordination are multifaceted and can stem from both intrinsic and extrinsic factors. The physiological basis of motion coordination is synergy, which is a key component throughout the entire motion process of the upper limbs and changes accordingly with different motion patterns. Motion coordination is specifically manifested as the precise control of the timing and spatial position of multi-degree-of-freedom movements during the movement process (temporal and spatial coordination), which is mainly dependent on the control of the nervous system. The internal neural control is further complemented by the coordination of the arms (inter-arm coordination) and body language (coordination of different limbs), which enables the coordinated movement of the limbs. Motion coordination of the upper limbs is a key component of the human motor system, which relies on the central nervous system and the cooperation of multiple muscles and joints, and involves fluidity, timing, and precision of movement, which is a challenge that is still not fully solved in the anthropomorphic motion planning system.

Classical neuromechanics suggests that the central nervous system relies on the interlocking of the muscular and skeletal systems to coordinate body movement, which is often called “synergy”. Recent research has revealed the existence of synergies at three levels, including kinematics, muscle mechanics, and neural centers (Bruton and O’dwyer, 2018), and has been widely applied to robotic arms to reproduce reaching movement (Liu et al., 2018) and grasping movement (Ficuciello et al., 2014) of the upper limbs. During human movement, the nervous system dynamically adjusts the synergies by regulating the control strategy to control the coordinated movement of the limbs to meet the requirements of the task. Hierarchical theory states that human high-level motion control units focus on generating upper limb configurations during reaching movement, and that low-level motor units synergistically control the joints associated with the movement to ensure coordination of upper limbs (Gosselin-Kessiby et al., 2008; Herbort and Butz, 2010). Correspondingly, by investigating the role of different synergy components in the reaching movement, Tang et al. (2019) found that the high percentage synergy is related to the movement trend, while the low percentage synergy is related to the specific task movements. Different principal components have some effects on the movement trajectory and endpoint accuracy, and the synergies are dynamically adjusted with different tasks. At the same time, the expressions of synergies in different motion patterns vary. Zhao et al. (2022) extracted the synergies under different numbers of trials and different arrival directions in point-to-point reaching movement experiments and found that the synergies increased with the number of trials or the number of arrival directions. When the number of experiments or the number of arrival directions reached a threshold, the synergies did not change significantly. The researchers hypothesized that different training patterns (number of trials, target category) affected muscle activation modules, which in turn affected synergies.

The study of synergistic movement of upper limbs is based on the foundation that humans activate discrete motion modules to perform biological activities through the cooperation and collaboration of different muscle groups to meet the needs of basic daily activities (Bizzi and Cheung, 2013). It is mainly categorized into three main components: synergistic control of nervous system (d’Avella, 2016), synergistic contraction of muscles (Pham et al., 2014; Tang et al., 2014), and synergistic movement of joints (Garcia-Rosas et al., 2018; Moiseev and Gorodnichev, 2022). The central nervous system receives information from the outside world as input, integrates and processes it to generate motor commands. The commands are transmitted and delivered, whose output is manifested as precise synergistic control of the relevant muscles and joints. The upper limb muscle groups take the received motor commands as input, then trigger the synergistic contraction of the corresponding muscles depending on the complexity of the commands, to produce the appropriate force to control skeletal movement. The synergistic contraction of muscles causes the attachment points on the corresponding bones to move, resulting in the simultaneous movement of multiple bones, which in turn causes synergistic movement of joints. The overall motion of the upper limbs is controlled by the motion of specific bones, whose specific path of motion is determined by the additional motion of specific joints. At the same time, the sensory and feedback mechanisms of the nervous system are able to provide timely information to the brain about the position, force, and movement status of the upper limbs, thus realizing more precise synergistic movement.

The study of synergistic movement of upper limbs is based on the foundation that humans activate discrete motor modules to perform biological activities through the cooperation and collaboration of different muscle groups to meet the needs of basic daily activities (Bizzi and Cheung, 2013). An important part of the synergistic movement of the upper limbs is the synergistic contraction of the muscles, which is accompanied by the synergistic movement of the muscles. Coscia et al. (2014) studied hand trajectories and shoulder and elbow angular displacement trajectories of an upper limb weight support in different horizontal planes, analyzed the synergy patterns of muscles, and found that modular organization activated by synergistic movement of muscle groups underlies upper limb reaching movement generation.

Synergistic movement of upper limbs also involve motion learning and memory processes. The human nervous system uses hundreds of millions of nerve cells to precisely regulate the body’s more than 600 muscles, turning flexion, extension, rotation, and grasping into functions that can run in the background without thinking. Through constant practice and repetitive movement, the brain can gradually build up the appropriate neural pathways and patterns to form a memory of muscle coordination.

Theoretical perspectives related to neuroscience and motion control suggest that the central nervous system views the multiple Dofs of the upper limbs as a luxury tool rather than a burden of control. In motion control of the human body, it is not necessary for the nervous system, which is the endpoint, to control all DoFs, which can lead to a lack of stability in the system. In motion coordination, stability and coordination do not coexist. To resolve this contradiction, Scholz and Schöner (1999) skillfully combine stability and coordination by designing experiments using a dynamical systems approach to approximate control structures in joint space. They proposed the uncontrolled manifold (UCM) hypothesis to quantify the joint coordination of human movement. Togo et al. (2016) proposed a UCM reference feedback control method that incrementally generated a target UCM from a given target end-effector trajectory and combined it with the target joint in joint space to minimize the cost function with respect to the input joint torque and torque variation. They also quantitatively compared the results of simulation and measurement experiments for a target tracking task. Statistical results showed that the proposed method quantitatively reproduced the kinematics and dynamics properties of the upper limbs (end-effector posture, end-effector velocity, and joint torque, etc.).

In upper limb rehabilitation, temporal and spatial coordination serve as an important indicator of whether the human body has normal motion ability, which directly reflects the rehabilitation effect of patients with physical disabilities. In complex scenarios such as industrial and service, temporal and spatial coordination can reflect the degree of collaboration of multiple robotic arms and directly affect the efficiency of task completion (Zhao et al., 2021). Gielniak et al. (2013) used motion clarity as a measure of a robot’s ability to understand human movement and engage in human-robot interaction, and used spatiotemporal coordination as a factor in synchronizing robotic arm movement with human movement in an anthropomorphic motion generation algorithm.

The aforementioned work is concerned with intra-arm coordination in single-arm movement, in addition to inter-arm coordination between dual-arm movement and coordination between the upper limbs and other parts of the body. Qu et al. (2019) constructed a learning model including PCA, GMM and GMR to extract the intra-arm and inter-arm coordination characteristics of the human upper limbs by analyzing the human bimanual motion data, derived the anthropomorphic coordination motion equations by combining the intra-arm and inter-arm coordination constraints, generated anthropomorphic trajectories of bimanual robots, and experimentally reproduced the anthropomorphic coordination motion, which could improve the human-robot interaction capability of the bimanual robots.

Furthermore, body language (gestures, body postures, facial expressions, etc.) is also an important part of conveying social information in human-computer interaction (Lütkebohle et al., 2010). Through body language, complemented by coordinated body movement to signal or imply goals, express emotions or intentions, and obtain status or feedback, human-computer interaction can be more natural and efficient. However, sometimes the information expected to be expressed by head movement is not perfect and the interacting objects cannot understand the full meaning, and then the auxiliary functions of other limbs become extremely important. Researchers have explored the role of coordination movement of different limbs (e.g., hand-eye coordination (Chao et al., 2018; Olson et al., 2020), head-eye coordination (Omrcen and Ude, 2010; Milighetti et al., 2011), neck-eye coordination (Rajruangrabin and Popa, 2010), etc.) in augmenting head movement at the level of information conveyance, as well as their planning and control schemes. Based on these studies, Zhang et al. (2015) proposed a new online generation method of anthropomorphic motion based on head-arm coordination, which considered not only the two-arm coordination motion, but also the head-arm coordination, and finally verified by computer simulation and physical experiments. Table 4 gives an overview of main approaches to solve motion coordination.

**TABLE 4:** Approaches to solve motion coordination.

Study	Task	Anthropomorphic criterion	Approach	Contribution
Rajruangrabin and Popa (2010)	Robot head human tracking	Eye-neck coordination	Visual feedback and optimization, reinforcement learning	Propose an optimization approach, combined with real-time visual feedback, to generate human-like motion
Milighetti et al. (2011)	Visual tracking of a moving target with unknown and arbitrary trajectory	Head-eye coordination	Adaptive Kalman Filter, trajectory tracking control	Proposed a gaze control scheme to achieve human-like joint motions
Coscia et al. (2014)	Reaching movements	Muscle synergies	Non-negative matrix factorization	Understand the effect of muscle coordination when performing upper extremity exercises
Zhang et al. (2015)	Tracking external targets and body parts	Head-arm coordination	A quadratic program-based method	Propose a novel head-arm-based human-like behavior generation scheme
Togo et al. (2016)	One-dimensional target-tracking task	Joint coordination	UCM	UCM reference feedback control can reproduce human-like joint coordination
Chao et al. (2018)	Saccade movements, hand spontaneous movements	Hand-eye coordination	Constructive neural networks	Build a reverse transformation from the robot actuators space to the robot visual space
Tang et al. (2019)	Reaching task	Kinematic synergies	PCA	Confirm that kinematic synergies can be used for exoskeleton motion planning
Qu et al. (2019)	Carrying and pouring	Intra-arm and inter-arm coordination	A learning model consisting of PCA, GMM and GMR	Propose a method based on human-arm coordination characteristics to enhance human-robot interaction ability
Zhao et al. (2022)	Point-to-point reaching movements	Muscle synergies	Non-negative matrix factorization	Promote applications of muscle synergies in clinical scenarios

Although researchers have proposed various coordination algorithms to control the coordinated motion of the robotic arm, due to the limitation of the understanding of the human movement control mechanism, the researches on motion coordination can only reduce the motion errors of the robotic arm in most cases, and cannot make it eliminated. By further studying the human-robot interaction mechanism and assisting more accurate and sensitive sensor technology to provide better theoretical guidance for the robot’s motion control strategy, it is possible to better optimize the coordination of the robot’s anthropomorphic motion.

## 5 Future challenges

With the in-depth study of human movement function, wearable exoskeleton robots (Liu et al., 2018) and medical robots (Zhang et al., 2020) with anthropomorphic motion planning ability have gradually come into view. In the future, it is expected that more products will be introduced to meet human needs. However, in order to provide high-quality services and realize large-scale applications, the following challenges need to be addressed based on existing researches.

(1) Intelligence and autonomy enhancement. In unstructured scenarios such as homes and restaurants, to provide better service, robots should be more intelligent, make fast and accurate decisions, and take appropriate actions based on task requirements and real-time situations to improve work efficiency. At the same time, the full autonomy of humanoid robots allows them to take on heavy, dangerous or boring tasks, which enables humans to focus more on creative and advanced thinking. Unfortunately, existing humanoid robots are not yet able to be fully autonomous from humans. With continuous advances in artificial intelligence, sensor technology, control algorithms, and other fields, we can expect future robots to achieve a higher level of autonomy.
(2) Multimodal interaction and human-robot fusion. To enhance the personalized interaction experience, the robot should integrate multiple sensors such as visual, auditory, and haptic (Li G. et al., 2019) to comprehensively understand the user’s behavioral patterns, accurately respond to the user’s needs, and monitor the user’s feedback. Through various forms of input and corresponding outputs, the multimodal interaction capability can realize a richer and more convenient human-computer interaction experience. However, current technologies cannot fully resolve the conflict between interaction efficiency and safety.
(3) Emotional interaction and emotional intelligence. Emotional interaction and emotional intelligence in humanoid robots enable them to better understand and respond to human emotional needs. Through emotional interaction, robots can communicate and interact emotionally with humans. Through emotional intelligence, robots can process and analyze emotional information and make corresponding intelligent decisions based on emotional information. The development of this technology will bring people more user-friendly and personalized robot services and support.
(4) Humanitarian and ethical considerations. The future development of humanoid robots should also focus on humanitarian and ethical considerations. Ethical guidelines must be followed in the design and application process to ensure that robots behave in accordance with moral and social values and are able to contribute positively to human wellbeing and social development.

## 6 Conclusion

In this article, we reviewed representative anthropomorphic motion planning researches for multi-degree-of-freedom robotic arms. By in-depth analysis of human natural motion, we proposed a novel classification method that incorporated human movement laws into robot motion control based on physiology, and constructed a more complete anthropomorphic planning system to better address the problem of anthropomorphic motion planning. This classification encompasses the majority of current anthropomorphic motor planning research results. It not only summarizes and integrates existing research results but also provides an in-depth exploration and understanding of the deeper causes of human movement ability. This categorization method comprehensively and systematically examines the reasons for the formation of unique human movement abilities in three major aspects: motion patterns, individual variation, and functional control. Firstly, from a physiological perspective, the formation of natural human movement ability is inextricably linked to body composition. The flexibility provided by the redundancy of the upper limbs ensures that humans can accomplish various types of complex tasks. Therefore, motion redundancy is the primary issue addressed in anthropomorphic motion planning. Secondly, individual variation is also a significant factor affecting human movement abilities. Each individual possesses unique physical characteristics, exercise habits, and psychological states, which can influence movement performance. Therefore, it is crucial to consider individual variation when designing anthropomorphic movement plans. Motion variation is a significant challenge in this domain. Finally, functional control is essential for human movement ability. The nervous system plays a pivotal role in regulating daily life movement. In addition, in order to maintain balance during movement and to improve the accuracy and stability of movement execution, the motion coordination of the limbs is an important symbol that distinguishes human beings from non-living beings (robots) or human beings with impaired motor function (patients with limb disabilities). Therefore, motion coordination is an important criterion for robot motion to be anthropomorphic. During the development, researchers have moved from single anthropomorphic criterion to consider multiple criteria to ensure that motion is sufficiently anthropomorphic. In addition, each section of the article discusses in detail the various research approaches to understanding the anthropomorphism of movement and expresses appreciations for the value that these findings provide in the anthropomorphic planning system. The article also points out the current challenges faced by anthropomorphic motion planning and suggests possible trends for the future. Once these difficulties are overcome, humanoid robots with more advanced anthropomorphic motion planning abilities will be realized in real life, contributing to the improvement of human living standards for the benefits of the society.
